# Vaccinating Adult Patients with Cirrhosis: Trends over a Decade in the United States

**DOI:** 10.1155/2016/5795712

**Published:** 2016-04-30

**Authors:** Abhijeet Waghray, Nisheet Waghray, Hicham Khallafi, K. V. Narayanan Menon

**Affiliations:** ^1^Department of Medicine, MetroHealth Medical Center/Case Western Reserve University, Cleveland, OH 44109, USA; ^2^Division of Gastroenterology/Hepatology, MetroHealth Medical Center/Case Western Reserve University, Cleveland, OH 44109, USA; ^3^Gastroenterology and Hepatology, Cleveland Clinic Foundation, Cleveland, OH 44195, USA

## Abstract

*Introduction*. The progression of chronic liver disease to cirrhosis involves both innate and adaptive immune system dysfunction resulting in increased risk of infectious complications. Vaccinations against pneumococcus, hepatitis A virus (HAV), and hepatitis B virus (HBV) are well tolerated and effective in disease prevention and reduction in morbidity and mortality. Prior studies assessing vaccination rates in patients with cirrhosis have specific limitations and to date no study has provided a comprehensive evaluation of vaccination rates in patients with cirrhosis in the United States.* Aim*. This study assessed vaccination rates for pneumococcus, HAV, and HBV in patients with cirrhosis.* Results*. Overall 59.7% of patients with cirrhosis received at least 1 vaccination during the study period. Vaccination rates within the same or following year of cirrhosis diagnosis were 19.9%, 7.7%, and 11.0% against pneumococcus, HAV, and HBV, respectively. Trend analysis revealed significant increases in vaccination rates for pneumococcus in all patients with cirrhosis and within subgroups based on age, gender, and presence of concomitant diabetes.* Conclusion*. The study demonstrated that vaccination rates in patients with cirrhosis remain suboptimal. Ultimately, the use of electronic medical record (EMR) reminders improved communication between healthcare professionals and public health programs to increase awareness are fundamental to reducing morbidity, mortality, and health-care related costs of vaccine preventable diseases in patients with cirrhosis.

## 1. Introduction

Cirrhosis is the 14th most common cause of death worldwide with over 33,000 deaths per year in the United States [1, 2]. The progression of chronic liver disease to cirrhosis involves both innate and adaptive immune system dysfunction recognized as cirrhosis-associated immune dysfunction (CAIDS) [3]. Decreased opsonic and reticuloendothelial (RE) cellular activity, complement levels, and neutrophil mobilization coupled with enhanced bacterial translocation underscore the multifactorial nature of immune dysfunction [4]. Portosystemic shunting further diminishes hepatic clearance of bacteria and endotoxins, placing cirrhotic patients who develop infection at an increased risk for sepsis, multiorgan failure, and death [5, 6]. Studies in cirrhotic patients also demonstrate a more pronounced inflammatory response to infection with elevated levels of TNF and IL-6 [7, 8]. Immune dysfunction in cirrhosis is characterized by both overstimulation and inhibition. Compared to overall infection rates of 5%–7% amongst hospitalized patients, bacterial infections occur in one-third of cirrhotic patients with the greatest risk (45%) in those admitted with GI bleeding [6, 9, 10]. The most common bacterial infections include spontaneous bacterial peritonitis (SBP), urinary tract infections, pneumonia, and sepsis with an unspecified source [10, 11]. Studies demonstrate that pneumonia accounts for 15%–20% of infections in patients with cirrhosis and is more likely to be associated with invasive pneumococcal pneumonia and systemic organ dysfunction [9–13]. Pneumonia also carries the highest 30- and 90-day mortality in patients with cirrhosis—an adjusted hazard ratio (aHR) of 2.95 and 2.57, respectively [14]. Therefore, vaccination guidelines for pneumonia are fundamental to preventing disease.

Like bacterial infections, studies have demonstrated that superinfection with hepatitis A (HAV) or B (HBV) in the setting of chronic liver disease or cirrhosis is associated with increased morbidity and mortality [15–18]. During the Shanghai epidemic in which over 300,000 people developed acute HAV, the case fatality rate was 5.6 times higher in those superinfected with HAV compared to individuals infected with HAV alone (0.05% versus 0.009%) [19, 20]. Confirming these findings, in a large series of 115,551 cases of acute HAV, 107 of 381 (28%) deaths were in patients with underlying chronic liver disease [21]. While superinfection with HAV has been associated with hepatic failure/mortality, accelerated progression to decompensated cirrhosis and hepatocellular carcinoma (HCC) has been linked to HBV coinfection [22, 23]. These studies demonstrate that superinfection with viral hepatitis is responsible for more pronounced and severe liver injury, making vaccination strategies for HAV and HBV critical.

Concomitant chronic diseases such as diabetes place cirrhotic patients at a high risk for complications [24–27]. Specifically, hyperglycemia and insulin resistance resulting from diabetes and cirrhosis are implicated in reduced chemotaxis and neutrophil/macrophage phagocytic function [28, 29]. It has been further demonstrated that, in chronic HCV, diabetes is an independent predictor for bacterial infections, encephalopathy, and hepatocellular carcinoma [30]. Thus, adults with coexisting chronic conditions may be more likely to develop complications from certain preventable diseases.

It is well established that adults with chronic conditions are at increased risk of vaccine preventable diseases resulting in prolonged hospitalizations and death. The role of immunization programs is critical and vaccines against pneumococcus, HAV, and HBV are safe, well tolerated, and highly effective [31–33]. Current recommendations from the American Association for the Study of Liver Diseases (AASLD) and Centers for Disease Control (CDC) endorse vaccination of all susceptible chronic liver disease patients [34, 35]. In 2008, the Centers for Medicare and Medicaid Services (CMS) adopted vaccination against HAV and HBV in patients with chronic HCV infection as a quality measure [36, 37]. In recent years, interest in preventive care and emphasis on meeting quality measures has intensified. There is a paucity of literature assessing vaccination rates in patients with cirrhosis and any generalization is limited by sample demographics and disease characteristics. No study to date has provided a comprehensive evaluation of vaccinations in patients with cirrhosis across the United States.

The aim of this study is to provide (1) an assessment of pneumococcal, hepatitis A (HepA), and hepatitis B (HepB) vaccination rates and a (2) cross-sectional comparison and trend analysis of vaccination rates in patients with cirrhosis over the last decade. Our patient sample is diverse including various practice types (primary care and specialists), demographic factors, and contact locations (inpatient and outpatient).

## 2. Methods

**Figure 1:**
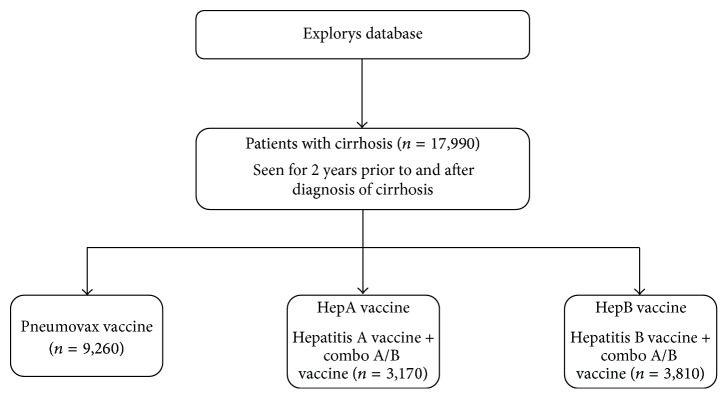
Vaccinations assessed in patients with cirrhosis.

All adult patients with a diagnosis of cirrhosis were identified in the Explorys database (ICD-9 codes: 571.2, 571.5) in June 2015. Patients were included in the study only if they were seen for at least 4 years (at least 2 years prior to the diagnosis of cirrhosis and at least 2 years after diagnosis). Patients were considered eligible for vaccination if titers for hepatitis A and B were not positive at time of cirrhosis diagnosis. Vaccination status was determined by CPT codes (pneumococcal vaccine: 90732, 90670; HepA vaccine: 90632; HepB vaccine: 90746, 90740, 90747), with a vaccination defined as receiving at least one dose of the vaccine during the study period. Patients that received the combination hepatitis A/B vaccination (CPT: 90636) were also included. Thus, 3 vaccinated groups of patients were established: those that received a (1) pneumococcal vaccine, (2) HepA vaccine (hepatitis A or combination of hepatitis A/B vaccine), and (3) HepB vaccine (hepatitis B or combination of hepatitis A/B vaccine) (Figure 1). Subgroup analysis was performed for vaccination rates in patients with diabetes mellitus (ICD-9: 250.xx).

Reported demographic data includes age, gender, race, and language spoken. The primary end point for the study was to determine the percentage of patients with cirrhosis that received a pneumococcal, HepA, or HepB vaccination at any given time. The percentage vaccinated is represented as total patients that received at least 1 vaccination during the study period divided by the total number of eligible patients with cirrhosis. Diagnosis dates were grouped by year (between 2004 and 2012) with vaccinations administered within the same year or following year of diagnosis included in trend analysis. Thus, vaccination rates were assessed between the years 2004 and 2013. Overall trends in vaccination rates were assessed for the defined period, in addition to subgroup analysis for gender (male or female), age (>65 or ≤65 years old), and coexistent diabetes.

Explorys is a deidentified standardized database (Explorys Inc., Cleveland, OH, USA) with an aggregate of over 44 million patients from healthcare systems across the United States. A variety of health information systems (electronic health records, laboratory, and billing systems) and data from inpatient and outpatient systems are used to compile the data in Explorys. Records are deidentified to meet standards by Health Insurance Portability and Accountability Act (HIPAA) and Health Information Technology for Economic and Clinical Health (HITECH) Act. Institutional Review Board (IRB) review was not required.

### 2.1. Statistical Analysis

Six groups of patients were formed based on vaccination status (vaccinated/not vaccinated) and type of vaccine administered (pneumococcus, HepA, or HepB vaccine). Demographic data as previously described was aggregated and expressed as frequencies and percentages. Quantitative variables were summarized as means. Cochran-Armitage test for trend assessed changes in vaccination rates between 2004 and 2013 across all vaccination types and subgroups (age, gender, and history of diabetes). A *P* value < 0.05 was considered significant and data were analyzed using JMP version 9.0 (SAS Institute Inc., Cary, NC, USA).

## 3. Results

A total of 17,990 patients with cirrhosis were identified in the Explorys system. Patients identified with cirrhosis were active within the system for at least 4 years as previously described. Complete demographic and clinical data are listed in Table 1. Fifty-four percent (*n* = 9,750) were male and 79.5% (*n* = 14,310) were Caucasian. Diabetes was common among cirrhotic patients in this cohort (50.8%; *n* = 9,130). Medicare was the major payer of healthcare for patients with cirrhosis (51.6%; *n* = 9,290). Overall 59.7% (*n* = 10,730) of patients received at least 1 vaccination against either pneumococcus (*n* = 9,260), HAV (*n* = 3,170), or HBV (*n* = 3,810) (Figure 1). Similar to the population with cirrhosis alone, 63.5% (*n* = 5,800) of patients with concurrent diabetes received at least 1 vaccination against pneumococcus, HAV, or HBV.

### 3.1. Trends in Vaccination

**Figure 2:**
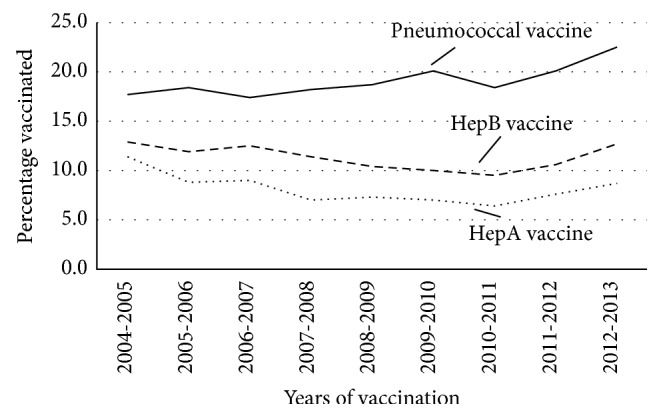
Percentage of total cirrhotics vaccinated between 2004 and 2013. Time represented as years of vaccination (same or following year of cirrhosis diagnosis).

**Figure 3:**
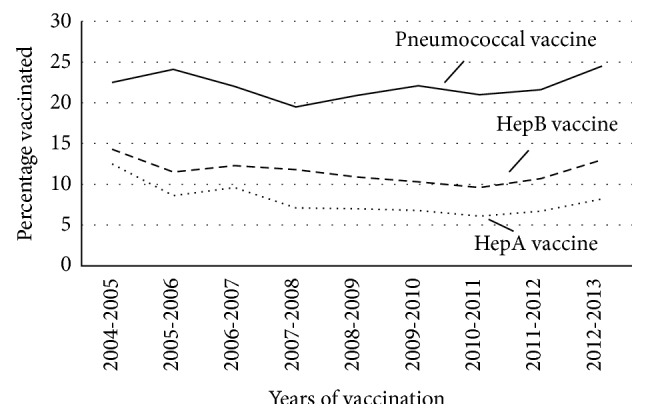
Percentage of cirrhotics with concurrent diabetes vaccinated between 2004 and 2013. Time represented as years of vaccination (same or following year of cirrhosis diagnosis).

Temporal trends for pneumococcus, HepA, and HepB vaccinations between 2004 and 2013 are shown in Figures 2 and 3. The data demonstrate that pneumococcal vaccination rates increased from 17.7% to 22.5% over the last decade (average of 20.0%; *P* < 0.01). On subgroup analysis for age, both patient groups, ≤65 and >65, had significant increases in the percentage of vaccinated patients (+5.4% and +3.8% increase, resp.; *P* < 0.01). While vaccination rates for pneumococcus increased amongst females with cirrhosis (*P* = 0.01), there was an even larger increase seen in male patients (+7.5%; *P* < 0.01). For cirrhotic patients with concurrent diabetes, vaccination rates for pneumococcus increased from 22.5% (2004-05) to 24.5% (2012-13) (*P* = 0.04).

Unfortunately, the gains seen in pneumococcus vaccination rates were not replicated with HepA or HepB. Vaccination rates for HepA were highest in patients diagnosed with cirrhosis in 2004 (11.4%) with no significant change in rates seen from 2004 to 2013 (average of 7.7%; *P* = 0.44). Further, trend analysis for the decade identified no significant change in vaccination rates for HepA among patients ≤ 65 (*P* = 0.43) and >65 (*P* = 0.90) years of age, males (*P* = 0.17) or females (*P* = 0.95). In patients with concurrent diabetes vaccination rates peaked within 2004-2005 (12.5%) with a significant reduction and nadir in 2010-2011 (6.1%; *P* = 0.04). Despite this initial decline, vaccination rates subsequently rebounded to 8.2% by 2012-2013. Trends in vaccination for HepB remained stable since 2004 for all patients with cirrhosis (average 11.0%; *P* = 0.61). Over this period, amongst males there was a reduction in vaccination rates by 3.1% (*P* = 0.05) compared to an increase in rates for females by 3.2% (*P* = 0.98), though neither was statistically significant. For patients > 65 years of age, vaccination rates significantly declined from 10.0% in 2004-2005 to a nadir of 6.3% in 2010-2011 but significantly increased to 9.7% by 2012-2013 (*P* < 0.01). No significant change in vaccination rates were seen for adults ≤ 65 years of age (*P* = 0.28). While vaccination rates for patient with concurrent diabetes ranged from 14.3% in 2004-2005 to 9.6% in 2010-2011 there was no significant difference noted (*P* = 0.89) (Figure 2).

## 4. Discussion

Described as a state of immune dysfunction, cirrhosis related changes are characterized by (1) reduced RE cells, (2) diminished immune response/phagocytic activity, and (3) portosystemic shunting [5, 6, 38, 39]. These factors limit the clearance of bacteria, endotoxins, and cytokines, making cirrhotic patients particularly susceptible to infections with related increases in morbidity and mortality. Thus, cirrhosis is a complex immunocompromised state, in which vaccination strategies are vital towards disease prevention and reduction of infection related morbidity and mortality.

Little is known regarding vaccination practices in patients with cirrhosis. While prior studies have demonstrated that vaccination rates remain low, they have been limited to specific vaccines and patient populations. In a 2011 study using the National Health and Nutrition Examination Survey (NHANES) database, Younossi and Stepanova assessed vaccination rates from 1999 to 2008 in chronic liver disease patients. Two groups, (1) 1999 to 2004 and (2) 2005 to 2008, were evaluated with increased vaccination rates against both HAV and HBV during the later period. Regardless, overall vaccination rates remained low (13.3% to 20.0% and 23.4% to 32.2%, resp.) [40]. A limitation of the NHANES database remains the risk of overestimating vaccination rates as hospitalized patients are not included. In our study, 84.3% of patients had at least 1 hospital visit and 86.8% had at least 1 office visit. Further, the NHANES data represents overall vaccination rates rather than vaccination from time of cirrhosis diagnosis. Two separate studies involving chronic HCV patients within the Veterans Affairs (VA) system also assessed vaccination rates or documented immunity to HAV (ranging from 46% to 71%) and HBV (ranging from 46% to 70%) [41, 42]. Wörns et al. further showed that, in patients with autoimmune hepatitis in Germany, vaccination rates of 13% and 11% were found for HAV and HBV vaccinations, respectively [43]. Interestingly, these three studies reveal a large variation in vaccination rates and it is likely that heterogeneity in etiology of chronic liver disease may explain these findings. Further, the VA population also represents a unique demographic of patients within the healthcare system, making generalization of findings difficult.

In our study, 59.7% of patients with cirrhosis received at least one vaccination for either pneumococcus, HAV, or HBV. Vaccination within the same or the following year of a diagnosis of cirrhosis accounted for less than half of all vaccinations given to cirrhotic patients for a specific vaccine. On average, only 19.8%, 7.7%, and 11.0% of patients received a pneumococcus, HepA, or HepB vaccine in the same or following year of cirrhosis diagnosis. Analysis of vaccination trends for pneumococcus (2004–2013) demonstrated a significant increase amongst all cirrhotic patients (+4.8%), with the greatest increase in male patients (+7.5%). Over the same period of time, no significant changes in vaccination rates were observed amongst HAV or HBV. Increased pneumococcal vaccination rates seen in our study may partly be attributed to current recommendations for universal vaccination in all patients with an increasing emphasis on this as a quality measure.

Patients with cirrhosis and concurrent diabetes are at increased risk of infection and related complications [24–27]. It is postulated that hyperglycemia and insulin resistance impair both the innate and adaptive immune system by diminishing chemotaxis, neutrophil/macrophage function, inhibiting activation of the complement cascade and cell mediated immunity [28, 29]. In patients with cirrhosis, diabetes remains an independent predictor of bacterial infections, confers an increased risk of hepatic failure, and is an independent negative predictor of survival [24, 30, 44, 45]. On trend analysis from 2004 to 2013, pneumococcal vaccination rates amongst patients with cirrhosis and concomitant diabetes increased by 2.0%, which was lower than in the general cirrhotic population. During the same period HepA vaccinations significantly declined by 4.3%, while there was no significant change in HepB vaccination rates. Overall, vaccination rates in this high risk cohort were less than 25%, leaving the majority of patients vulnerable to otherwise preventable diseases.

This study highlights several issues related to the role of guidelines and how best to implement vaccination strategies. The literature shows that vaccination against preventable diseases such as pneumococcus, HAV, and HBV reduces morbidity, mortality, and overall healthcare costs. While the safety and tolerability of vaccinations in chronic liver disease appear to be well documented, immune response to vaccines in advanced liver disease has been called into question [46–50]. Smallwood et al. demonstrated that immunogenicity to vaccinations varied with the degree of hepatic decompensation, where patients with more severe disease were less likely to seroconvert [50]. These studies underscore the importance of pursuing vaccination strategies early in the course of chronic liver disease.

In the current healthcare system there is an increased emphasis placed on the delivery of cost effective preventative care [51]. Healthcare providers including specialists, primary care physicians, nurses, and advanced nurse practitioners are in a unique position to address concerns related to vaccines, a source of noncompliance with vaccination recommendations. Discussions regarding the safety and importance of vaccinations in chronic liver disease will help to promote awareness and implementation of successful vaccination programs. Enhancing awareness through public health programs and the use of social media may reduce the risk from vaccine preventable diseases. In addition to public programs, improving communication between primary care providers and specialists, and defining responsibility for immunizations, is critical. In a retrospective analysis, Jacobs et al. demonstrated that patients with chronic liver disease were more likely to receive influenza and pneumococcal vaccinations from their primary care physician, while hepatitis A or B vaccinations were more likely to be provided by specialists [52]. Uncertainty amongst providers and their role can be addressed through communication tools in electronic medical records (EMRs) and “best practice” electronic reminders. Loo et al. demonstrated then when clinicians were reminded via EMR, there was a 12.5% increase in patients that received a pneumococcal vaccine [53]. Further supporting the role of EMR reminders are improved performance measures seen in other chronic diseases such as diabetes [54]. Ultimately, prospective studies will be required to assess whether EMR and “best practice” reminders improve adherence to vaccination guidelines.

There are several limitations to our study. This is a retrospective study with reliance on diagnostic and procedural coding that cannot be validated for an individual patient. Further, our study focused on assessing the percentage of patients that received a vaccination. Thus, a subgroup of patients may have been vaccinated but did not develop immunity. Limitations of the database also do not allow tracking completion of a required vaccination series. Thus, our estimates for HepA/HepB vaccination likely represent an overestimation of actual immunity. Finally, because this is a review of database information, intention of a physician to vaccinate or patient compliance issues cannot be adequately assessed. Despite these limitations, this study encompassed a broad spectrum of patients with varying demographic characteristics from inpatient and outpatient encounters, thus representing the most comprehensive assessment of vaccination status in cirrhotic patients.

In summary, this study demonstrates that vaccination rates for pneumococcus, HepA, and HepB in patients with cirrhosis remain suboptimal. Even amongst a high risk cohort, such as those with concomitant diabetes, vaccination rates were less than 25%. Successful public health programs, implementation of “best practice” electronic reminders, and communication between specialists and primary care providers are fundamental to addressing the very issues that plague vaccination adherence rates. Clearly, this study demonstrates that more needs to be done to reduce morbidity, mortality, and health-care related costs of vaccine preventable diseases in patients with cirrhosis.

## Figures and Tables

**Table 1:** Demographics for patients with cirrhosis.

Demographics	
Age, *n* (%)	
20–65	11,440 (63.6)
Greater than 65	6,550 (36.4)
Gender, *n* (%)	
Male	9,750 (54.2)
Insurance status, *n* (%)	
Medicare	9,290 (51.6)
Race, *n* (%)	
Caucasian	14,310 (79.5)
African American	3,040 (16.9)
Comorbid conditions, *n* (%)	
Diabetes	9,130 (50.8)
Language spoken, *n* (%)	
English	16,790 (93.3)
Spanish	400 (2.2)
